# Residual Risk of Coronary Atherosclerotic Heart Disease and Severity of Coronary Atherosclerosis Assessed by ApoB and LDL-C in Participants With Statin Treatment: A Retrospective Cohort Study

**DOI:** 10.3389/fendo.2022.865863

**Published:** 2022-04-20

**Authors:** Tianci Yao, Weilin Lu, Jinshan Ke, Hao Zhang, Xiaofang Zhao, Bei Song, Ting Liu, Qinmei Ke, Chengyun Liu

**Affiliations:** ^1^Department of Geriatrics, Union Hospital, Tongji Medical College, Huazhong University of Science and Technology, Wuhan, China; ^2^Department of Clinical Laboratory, Shanghai Hudong Hospital, Shanghai, China

**Keywords:** statin, ApoB, LDL-C, coronary atherosclerotic heart disease, residual risk, coronary atherosclerosis, syntax scores

## Abstract

**Background:**

Low-density lipoprotein cholesterol (LDL-C) is the primary target of lipid-lowering therapy on the management of hypercholesterolemia in the United States and European guidelines, while apolipoprotein B (apoB) is the secondary target. The objective was to determine if elevated levels of apoB is superior to LDL-C in assessing residual risk of coronary atherosclerotic heart disease and severity of coronary atherosclerosis in participants with statin treatment.

**Methods:**

This study included 131 participants with statin treatment. The generalized linear model and relative risk regression (generalized linear Poisson model with robust error variance) were used to analyze the association of the levels of apoB and LDL-C with the severity of coronary atherosclerosis and residual risk of coronary atherosclerotic heart disease.

**Results:**

Categorizing apoB and LDL-C based on tertiles, higher levels of apoB were significantly associated with the severity of coronary atherosclerosis (*P*_trend_ = 0.012), whereas no such associations were found for elevated levels of LDL-C (*P*_trend_ = 0.585). After multivariate adjustment, higher levels of apoB were significantly associated with residual risk of coronary atherosclerotic heart disease. When compared with low-level apoB (≤0.66 g/L), the multivariate adjusted RR and 95% CI of intermediate-level apoB (0.67–0.89 g/L) and high-level apoB (≥0.90 g/L) were 1.16 (1.01, 1.33) and 1.31 (1.08, 1.60), respectively (*P*_trend_ = 0.011). There was a 45% increased residual risk of coronary atherosclerotic heart disease per unit increment in natural log-transformed apoB (*P*_trend_ <0.05). However, higher levels of LDL-C were not significantly associated with residual risk of coronary atherosclerotic heart disease. When compared with low-level LDL-C (≤1.56 mmol/L), the multivariate adjusted RR and 95% CI of intermediate-level LDL-C (1.57–2.30 mmol/L) and high-level LDL-C (≥2.31 mmol/L) were 0.99 (0.84, 1.15) and 1.10 (0.86, 1.42), respectively (*P*_trend_ = 0.437). Similar results were observed in the stratified analyses and sensitivity analyses. No significant interactions were detected for both apoB and LDL-C (all *P*_interaction_ *>*0.05).

**Conclusions:**

Elevated apoB are superior in assessing the residual risk of coronary atherosclerotic heart disease and severity of coronary atherosclerosis in participants with statin treatment.

## Introduction

With the improvement of living standards and the accelerated aging of the population in China, the incidence of coronary atherosclerotic heart disease has increased dramatically. Dyslipidemia as an independent risk factor for coronary artery disease has drawn widespread attention. A previous study predicted that elevated serum cholesterol levels will lead to an additional 9.2 million coronary atherosclerotic heart disease in China between 2010 and 2030 (1). Studies showed that lowering levels of serum low-density lipoprotein cholesterol (LDL-C) can significantly reduce the risk of coronary artery disease (2), and LDL-C is the primary target of lipid-lowering therapy in the United States and European guidelines in hypercholesterolemia management (3, 4). Guidelines focus on lowering LDL-C concentration to reduce atherosclerotic cardiovascular disease (ASCVD) risk. However, numerous clinical trials of statin and non-statin therapy showed persistent residual ASCVD risk despite aggressive LDL-C lowering (5–7), suggesting other atherosclerotic lipoproteins need to be considered. Identifying residual risk of coronary atherosclerotic heart disease in populations with low levels of LDL-C is essential for the prevention of ASCVD.

At present, more factors are increasingly considered to be associated with residual risk of ASCVD after LDL-C lowering. For example, Triglyceride-rich lipoproteins (TGRLs) and lipoprotein(a) have been shown to be associated with residual risk in patients treated to low concentrations of LDL-C (8, 9). Apolipoprotein B (ApoB) is the main protein constituent of atherogenic lipoproteins, namely, very low-density lipoprotein (VLDL), intermediate density lipoprotein (IDL), lipoprotein(a) (Lp(a)), and LDL-C, and each atherogenic lipoprotein particle contains one molecule of apoB, so the concentration of apoB is proportional to the total number of atherogenic lipoprotein particles (10, 11). The accumulation of apoB under the endothelium was confirmed to be a key initiating event of atherosclerosis (12, 13). A study found that elevated levels of apoB, but not LDL-C, were associated with an increased risk of myocardial infarction in participants with statin treatment (14). However, this study only included people of white ancestry. To explore whether apoB is superior to LDL-C in assessing residual risk of coronary atherosclerotic heart disease in the Chinese population with statin treatment, and to further investigate whether the concentrations of apoB and LDL-C are associated with the severity of coronary atherosclerosis in participants with statin treatment, we conducted a retrospective cohort study. We calculated the syntax scores according to the invasive coronary angiography, and the syntax scores was used to evaluate the severity of coronary atherosclerosis.

## Materials and Methods

### Study Population

**Figure 1:**
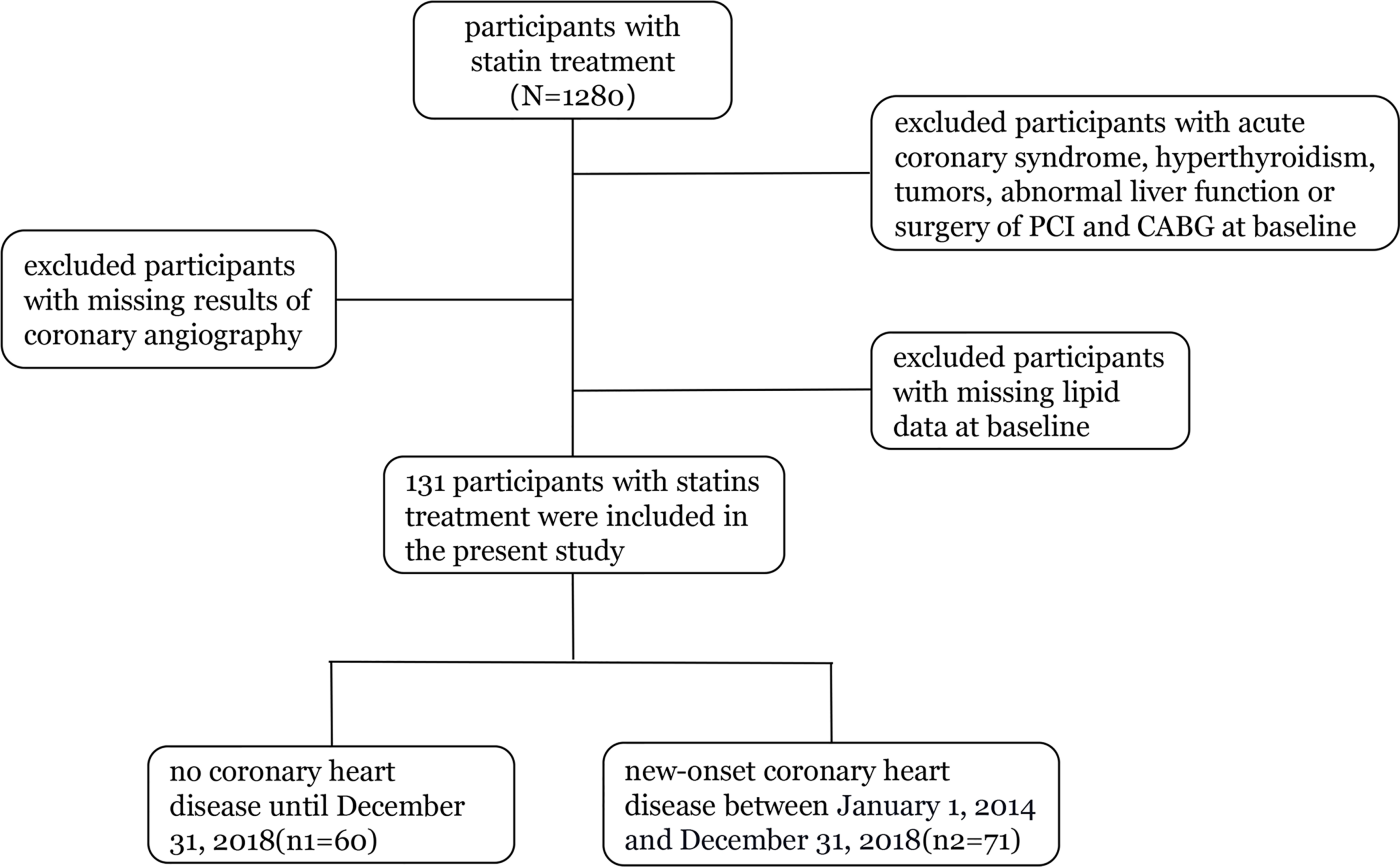
Flow diagram of participants enrolled.

Our study included 1,280 statin-treated participants with measurements for apoB and LDL-C at baseline. We excluded participants with acute coronary syndrome, hyperthyroidism, tumors, abnormal liver function or surgery of PCI and CABG at baseline, those with missing lipid data at baseline, and persons with missing results of coronary angiography during follow-up (2014 to 2018). The final cohort for analysis included 131 participants (**Figure 1**).

### Measurements of Lipid

We measured the concentration of total cholesterol, triglycerides, and high-density lipoprotein (HDL) cholesterol using colorimetric assays. When triglycerides were below 4 mmol/l, Friedewald equation was used to estimate the LDL-C concentration (LDL cholesterol = total cholesterol − HDL cholesterol – triglycerides/2.2 in mmol/l), and otherwise it was measured directly. ApoB, apolipoprotein A1 (apoA1), and lipoprotein(a) were measured using turbidimetric assays.

### Assessment of Covariates

Information on age, sex, smoking status, disease status, and medication use were collected from the hospital inpatient system. Hypertension was defined as taking antihypertensive medication, systolic BP ≥140 mmHg, or diastolic BP ≥90 mmHg (15). Diabetes was defined as taking antidiabetes drugs, injecting insulin, fasting blood glucose ≥7.0 mmol/L, 2-h plasma glucose (PG) ≥200 mg/dl (11.1 mmol/L) during OGTT or glycated hemoglobin (HbA1c) ≥6.5% (16). Venous blood samples were collected in the morning after an overnight fast and were processed within 2 h. Invasive coronary angiography was performed by experienced interventional doctor blinded to the data of subjects.

### Outcomes

The primary outcome of the study was new-onset coronary atherosclerotic heart disease between January 1, 2014, and December 31, 2018. In patients receiving lipid-lowering therapy, we assessed residual risk of coronary atherosclerotic heart disease based on new-onset coronary atherosclerotic heart disease. Invasive coronary angiography showed more than 50% luminal diameter narrowing in at least one major coronary artery by two experienced interventional cardiologists, and coronary atherosclerotic heart disease can be diagnosed. The secondary outcome was the severity of coronary atherosclerosis, also based on invasive coronary angiography. The syntax scores were used to quantitatively evaluate the severity of coronary atherosclerosis (17). The syntax scores can comprehensively and quantitatively evaluate the complex anatomical characteristics of the coronary arteries, namely, the location, severity, bifurcation, and calcification of the coronary arteries (18, 19). According to the invasive coronary angiography, we calculated the syntax scores on the website (www.syntaxscore.com), and another researcher calculated the score again a week later. The results of the two calculations were basically the same.

### Statistical Analysis

Categorizing concentrations of apoB and LDL-C were based on tertiles. The baseline characteristics of the study population were described according to apoB and LDL-C concentrations. The differences among groups were analyzed using the Chi-squared test for categorical variables, expressed as absolute frequency (%). For continuous variables, one-way analysis of variance or Kruskal–Wallis test were used to analyze the differences among groups, expressed as mean ± standard deviation (SD).

The generalized linear model was also used to detect the association of the levels of apoB and LDL-C with the severity of coronary atherosclerosis (evaluated by the syntax scores), covariates, namely, age, sex, diabetes, hypertension, smoking status, total cholesterol, triglycerides, apolipoprotein A1, and lipoprotein(a) were adjusted. There was no multicollinearity (defined as a correlation r ≥0.8 between variables) between apoB or LDL-C and adjusted covariates. Relative risk regression (generalized linear Poisson model with robust error variance) was used to estimate relative risk (RR) and 95% CIs for the association of the level of apoB and LDL-C with the residual risk of coronary atherosclerotic heart disease. According to tertiles, the levels of apoB and LDL-C were categorized into three groups: low-level apoB (≤0.66 g/L), moderate-level apoB (0.67–0.89 g/L), and high-level apoB (≥0.90 g/L); low-level LDL-C (≤1.56 mmol/L), moderate-level LDL-C (1.57-2.30mmol/L), and high-level LDL-C (≥2.31 mmol/L). The levels of apoB and LDL-C were also analyzed as a continuous variable after natural log transformation. In the multivariate models, we did not adjust any variables in model 1. In model 2, we adjusted for age (years), sex (female or male), smoking status (never smoker or smoker), diabetes (no or yes), and hypertention (no or yes). In model 3, we further adjusted for total cholesterol, triglycerides, apolipoprotein A1, and lipoprotein(a). Testing for linear trends was by assigning a median value to each category as a continuous variable.

Stratified analyses were also conducted by age (<65 or ≥65 years), sex (female or male), smoking status (never smoker or smoker), diabetes (no or yes), and hypertention (no or yes). The *P*-values for the product terms between level of apoB or LDL-C and stratification variables were used to estimate the significance of interactions. In order to further test the robustness of the research findings, we conducted several sensitivity analyses. First, logistic regression was used to estimate odds ratios (OR) and 95% CIs for the association of levels of apoB or LDL-C with the residual risk of coronary atherosclerotic heart disease. Second, the concentration of lipids may be affected by renal function; renal function assessed by estimated glomerular filtration rate (calculated by using the Chronic Kidney Disease Epidemiology Collaboration formula) was further adjusted (20). Third, we analyzed whether the association would change if only individuals with higher syntax scores were selected. All of the analyses were conducted using SPSS (version 26.0). Two-sided *P <*0.05 was considered statistically significant.

## Results

### Baseline Characteristics of Participants With Statins Treatment

A total of 131 participants (mean age, 64.98 years; 57.25% male) with statins treatment were included in the present study. The mean (95% CI) concentration of apoB and LDL-C at baseline was 0.81 (0.76, 0.85) g/L and 2.04 (1.90, 2.18) mmol/L. The baseline characteristics of the study participants according to apoB concentration are shown in **Table 1**. No significant differences were found in baseline characteristics, namely, age, sex, diabetes, hypertension, and smoking status. Serum lipids, namely, total cholesterol, triglycerides, and LDL-C differed significantly according to concentration of apoB. **Supplementary Table 1** shows the baseline characteristics of study participants based on LDL concentration.

**Table 1:** Baseline characteristics of participants with statins treatment according to ApoB concentrations.

	ApoB concentrations (g/L)				
	Total	≤0.66	0.67–0.89	≥0.90	*P*_trend_
Baseline characteristics					
Age(years)	64.98 ± 7.68	65.66 ± 7.44	64.36 ± 8.13	64.91 ± 7.55	0.647
Sex					
Female	56 (42.7%)	21 (47.7%)	16 (36.4%)	19 (44.2%)	0.734
Male	75 (57.3%)	23 (52.3%)	28 (63.6%)	24 (55.8%)	
Diabetes					
No	83 (63.4%)	25 (56.8%)	27 (61.4%)	31 (72.1%)	0.141
Yes	48 (36.6%)	19 (43.2%)	17 (38.6%)	12 (27.9%)	
Hypertention					
No	40 (30.5%)	13 (29.5%)	11 (25.0%)	16 (37.2%)	0.443
Yes	91 (69.5%)	31 (70.5%)	33 (75%)	27 (62.8%)	
Smoking status					
Never smoker	85 (64.9%)	32 (72.7%)	29 (65.9%)	24 (55.8%)	0.100
Smoker	46 (35.1%)	12 (27.3%)	15 (34.1%)	19 (44.2%)	
Lipids					
TC (mmol/L)	4.18 ± 1.18	3.45 ± 1.08	3.94 ± 0.53	5.16 ± 1.11	<0.001
TG (mmol/L)	1.73 ± 2.46	1.71 ± 3.62	1.42 ± 0.58	2.07 ± 2.16	<0.001
HDL-C (mmol/L)	1.18 ± 0.31	1.18 ± 0.37	1.15 ± 0.31	1.21 ± 0.23	0.143
ApoA1 (g/L)	1.26 ± 0.25	1.26 ± 0.25	1.25 ± 0.25	1.27 ± 0.24	0.945
Lp(a) (mg/dl)	23.94 ± 26.69	20.58 ± 23.09	20.97 ± 21.21	30.41 ± 33.68	0.159
LDL-C (mmol/L)	2.04 ± 0.81	1.39 ± 0.53	1.95 ± 0.49	2.79 ± 0.68	<0.001

Data expressed as absolute frequency (%) and mean ± SD.

ApoB was categorized into three groups: low-level (≤0.66 g/L), moderate-level (0.67–0.89 g/L), high-level (≥0.90 g/L).

### Association of ApoB and LDL-C With Severity of Coronary Atherosclerosis

**Figure 2:**
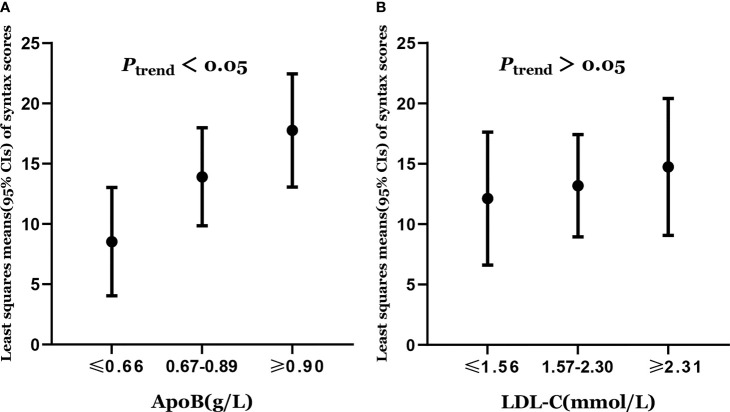
**(A)** Least squares means of syntax scores was estimated according to the levels of apoB. **(B)** Least squares means of syntax scores was estimated according to the levels of LDL-C. Dots represents the least squares means of syntax scores, up and down lines respectively represent the upper and lower limits of the 95% confidence interval for the least squares means. The least squares means of syntax scores was estimated using generalized linear model with adjustment of age (years), sex (female or male), smoking status (never smoker or smoker), diabetes (no or yes), hypertention (no or yes), total cholesterol, triglycerides, apolipoprotein A1, and lipoprotein (a).

Least squares means of syntax scores was estimated according to the levels of apoB and LDL-C. The least squares means and 95% CI of syntax scores from lowest to highest apoB categories (≤0.66, 0.67–0.89, and ≥0.90 g/L) were 8.53 (4.04, 13.02), 13.90(9.84, 17.97), and 17.75 (13.06, 22.44), respectively (**Figure 2A**). Higher levels of apoB were significantly associated with the severity of coronary atherosclerosis (*P*_trend_ = 0.012) (**Table 2**). However, higher levels of LDL-C were not significantly associated with the severity of coronary atherosclerosis (*P*_trend_ = 0.585) (**Table 2**). The least squares means and 95% CI of syntax scores from lowest to highest LDL-C categories (≤1.56, 1.57–2.30, and ≥2.31 mmol/L) were 12.12 (6.61, 17.62), 13.18 (8.94, 17.41), and 14.74 (9.07, 20.40), respectively (**Figure 2B**).

**Table 2:** B (95%CIs) for the levels of apoB and LDL-C.

ApoB (g/L)	≤0.66	0.67-0.89	≥0.90	*P*_trend_
	1,00	5.37 (0.12, 10.63)	9.22 (2.14, 16.31)	0.012
LDL-C (mmol/L)	≤1.56	1.57-2.30	≥2.31	*P*_trend_
	1.00	1.06 (−4.87, 6.98)	2.62 (−6.78, 12.02)	0.585

B (95%CIs) for the levels of apoB and LDL-C was estimated using generalized linear model with adjustment of age (years), sex (female or male), smoking status (never smoker or smoker), diabetes (no or yes), hypertention (no or yes), total cholesterol, triglycerides, apolipoprotein A1, and lipoprotein (a).

### Association of ApoB and LDL-C With Residual Risk of Coronary Atherosclerotic Heart Disease

In total, 71 new coronary atherosclerotic heart disease cases were identified. After multivariate adjustment, higher apoB levels were significantly associated with residual risk of coronary atherosclerotic heart disease. When compared with low-level apoB (≤0.66 g/L), the multivariate adjusted RR and 95% CI of intermediate-level apoB (0.67–0.89 g/L) and high-level apoB (≥0.90 g/L) were 1.16 (1.01, 1.33) and 1.31 (1.08, 1.60), respectively, for the residual risk of coronary atherosclerotic heart disease (*P*_trend_ = 0.011) (**Table 3**). There was a 45% increased residual risk of coronary atherosclerotic heart disease per unit increment in natural log-transformed apoB (*P*_trend_ *<*0.05) (**Table 3**). However, higher LDL-C levels were not significantly associated with residual risk of coronary atherosclerotic heart disease. When compared with low-level LDL-C (≤1.56 mmol/L), the multivariate adjusted RR and 95% CI of intermediate-level LDL-C (1.57–2.30mmol/L) and high-level LDL-C (≥2.31 mmol/L) were 0.99 (0.84, 1.15) and 1.10 (0.86, 1.42), respectively, for the residual risk of coronary atherosclerotic heart disease (*P*_trend_ = 0.437) (**Table 3**).

**Table 3:** RR (95% CIs) for residual risk of coronary atherosclerotic heart disease according to levels of apoB and LDL-C.

ApoB (g/L)	≤0.66	0.67–0.89	≥0.90	*P*_trend_	Per one-unit increment in natural log-transformed apoB
Model 1	1.00	1.11 (0.96, 1.29)	1.20 (1.04, 1.38)	0.017	1.25 (1.04, 1.50)
Model 2	1.00	1.11 (0.97, 1.27)	1.21 (1.06, 1.39)	0.008	1.29 (1.06, 1.56)
Model 3	1.00	1.16 (1.01, 1.33)	1.31 (1.08, 1.60)	0.011	1.45 (1.06, 1.97)
LDL-C (mmol/L)	≤1.56	1.57–2.30	≥2.31	*P*_trend_	Per one-unit increment in natural log-transformed LDL-C
Model 1	1.00	0.95 (0.82, 1.10)	1.08 (0.93, 1.25)	0.265	1.10 (0.95, 1.28)
Model 2	1.00	0.96 (0.84, 1.11)	1.08 (0.93, 1.26)	0.294	1.11 (0.96, 1.28)
Model 3	1.00	0.99 (0.84, 1.15)	1.10 (0.86, 1.42)	0.437	1.26 (0.95, 1.67)

Model 1: no variables are adjusted.

Model 2: adjusted for age (years), sex (female or male), smoking status (never smoker or smoker), diabetes (no or yes), and hypertention (no or yes).

Model 3: further adjusted for total cholesterol, triglycerides, apolipoprotein A1 and lipoprotein (a).

Stratified analyses by age, sex, smoking status, diabetes, and hypertension observed consistent results (**Supplementary Tables 2** and **3**). No significant interactions were detected for both apoB and LDL-C (all *P*_interaction_ *>*0.05). In the sensitivity analyses, consistent results were demonstrated when logistic regression was used to estimate odds ratios (OR) and 95% CIs for the association of the level of apoB and LDL-C with the residual risk of coronary atherosclerotic heart disease (**Supplementary Table 4**). Similar results were detected after further adjusting for estimated glomerular filtration rate (**Supplementary Table 5**). The results were similar when we selected individuals with higher syntax scores (**Supplementary Table 6**).

## Discussion

Our study provides insights into the value of apoB versus LDL cholesterol in participants with statin treatment for identifying residual risk of coronary atherosclerotic heart disease and severity of coronary atherosclerosis. Higher levels of apoB were significantly associated with residual risk of coronary atherosclerotic heart disease and severity of coronary atherosclerosis in statin-treated participants, whereas no such associations were found for elevated levels of LDL-C. Therefore, elevated apoB are superior in assessing residual risk of coronary atherosclerotic heart disease and severity of coronary atherosclerosis in patients with statin treatment. Identifying residual risk of coronary atherosclerotic heart disease in statin-treated patients with low levels of LDL-C is essential for the prevention of coronary atherosclerotic heart disease. Our results suggest that apoB can be used to guide further treatment in statin-treated patients with low levels of LDL-C.

The most likely explanation for our results is that apoB includes the atherogenic risk due to the TG-rich VLDL apoB particles and the cholesterol-rich LDL apoB particles (21), but LDL-C ignores the atherogenic potential of TG-rich lipoproteins. The concentration of apoB is proportional to the total number of atherogenic lipoprotein particles. Studies found that the trends of apoB and LDL-C differs in more than one quarter of people, especially in people with metabolic risk factors (such as obesity or type 2 diabetes) (22) and those taking statins (23). The reason for the inconsistent trends in the levels of apoB and LDL-C is that LDL-C is lowered relatively more than cholesterol of other apoB-containing lipoprotein (24).

Similar results have been reported in previous studies on participants with statin treatment. In the Air Force/Texas Coronary Atherosclerosis Prevention Study (AFCAPS/TexCAPs), apoB concentration was significantly associated with future cardiovascular events after one year of treatment, but not LDL-C (25). Kastelein et al. pooled the TNT and IDEAL studies, and found that apoB were more closely associated with cardiovascular events than levels of LDL-C in patients with statin treatment (26). Ference et al. found that the risk of cardiovascular events was proportional to the attenuated reduction in apoB, but significantly less than per unit change in LDL-C. The clinical benefit of lowering LDL-C levels may depend on the corresponding reduction in apoB-containing lipoprotein (27). In another study of Ference, the association of the levels of triglycerides and LDL-C with the risk of coronary heart disease was proportional to the absolute change in apoB. In multivariable Mendelian randomization analyses, the associations of levels of triglyceride and LDL-C with the risk of coronary heart disease became null after adjusting for apoB (28). However, while none of these studies included Chinese, our study shows that apoB is also superior to LDL-C in assessing residual risk of coronary atherosclerotic heart disease in the Chinese population with statin treatment.

In multiple previous studies, apob and LDL-C were associated with the severity of coronary atherosclerosis, the severity of coronary atherosclerosis was estimated based on the number of coronary artery lesions or Gensini score, and the study population was patients with suspected coronary heart disease or untreated patients (29–31). We conducted this research to determine whether the concentrations of apoB and LDL-C are still associated with the severity of coronary atherosclerosis in participants with statin treatment. Both Gensini score and syntax score are used to quantitatively evaluate the severity of coronary atherosclerosis based on the results of invasive coronary angiography. The Gensini score calculates the lesion score according to the lesion location and the degree of stenosis. Syntax score not only includes the lesion location and the degree of stenosis, but also further considers the bifurcation, calcification, and thrombus of coronary. Studies have confirmed that the syntax score was superior to the Gensini score in assessing the severity of coronary atherosclerosis (32). Our study is the first to show that concentrations of apoB are associated with the severity of coronary atherosclerosis (evaluated by syntax scores) in participants with statin treatment, but not in LDL-C.

Traditional epidemiological methods such as prospective studies and randomized controlled trials have confirmed that cholesterol (especially LDL cholesterol) and triglycerides are risk factors of cardiovascular disease, and elevated levels of cholesterol and triglyceride can significantly increase the risk of cardiovascular disease (33–35). However, many participants still had the residual risk of cardiovascular disease after LDL-C lowering (5–7), cardiovascular disease may be caused by a series of complex factors. For example, statins tend to increase Lp(a) levels, possibly contributing to the residual risk of cardiovascular disease, and lowering plasma Lp(a) levels can significantly decrease the residual risk of cardiovascular disease (36). Residual risk of cardiovascular disease was also associated with elevated plasma triglycerides and abnormal metabolism of triglyceride-rich lipoproteins (TRLs) (9).

At present, LDL-C is still the primary target of lipid-lowering therapy on the management of hypercholesterolemia in the United States guidelines and the European guidelines (3, 4). The 2019 European Society of Cardiology/European Atherosclerosis Association guidelines emphasize routine measurement of apoB, whereas the US guidelines do not. Compared with the US guidelines, the European guidelines highlight the status of apoB. Analyzing the reasons for this, there is ample evidence that lowering apoB levels significantly reduces the risk of coronary heart disease. However, the current evidence on the threshold for apoB as a risk modifier in patients with statin treatment is relatively insufficient, and further research is required (14). Our results suggest that elevated apoB is superior in assessing the residual risk of coronary atherosclerotic heart disease and severity of coronary atherosclerosis in patients with statin treatment. Thus, in patients receiving lipid-lowering therapy, apoB may be considered for guiding further treatment intensification even if LDL cholesterol is low. Routine measurement of apoB is recommended.

## Conclusions

We observed significant associations of higher levels of apoB with residual risk of coronary atherosclerotic heart disease and severity of coronary atherosclerosis in statin-treated participants, but not in LDL-C. Our results suggested that elevated apoB are superior in assessing residual risk of coronary atherosclerotic heart disease and severity of coronary atherosclerosis in participants with statin treatment.

## Study Limitations

First of all, this is a single-center retrospective cohort study, so it is necessary to design a multi-center prospective study to further verify our conclusions. Then it is possible that lacking information on types and doses of statins will induce bias, and future studies with information on types and doses of statins are encouraged.

## Data Availability Statement

The raw data supporting the conclusions of this study will be available from the corresponding author on reasonable requests.

## Ethics Statement

The study protocol was reviewed and approved by the Institutional Review Committee of Union Hospital Affiliated to Tongji Medical College of Huazhong University of Science and Technology, and conforms to the concept of the Declaration of Helsinki and its amendments. We verbally informed the participants that the data will be used for medical research anonymously. No informed consent was signed for this study in accordance with the national legislation and the institutional requirements.

## Author Contributions

TY, QK and CL conceived and designed the study. TY and WL jointly responded to the editor and reviewers and revised manuscript. TY and JK analyzed the data and wrote the first draft of the manuscript. WL, HZ, XZ, BS and TL collected data. QK and CL revised this manuscript. All authors had access to study data and approved the decision to submit the manuscript.

## Funding

This research was supported by the National Natural Science Foundation of China (No. 81974222).

## Conflict of Interest

The authors declare that the research was conducted in the absence of any commercial or financial relationships that could be construed as a potential conflict of interest.

## Publisher’s Note

All claims expressed in this article are solely those of the authors and do not necessarily represent those of their affiliated organizations, or those of the publisher, the editors and the reviewers. Any product that may be evaluated in this article, or claim that may be made by its manufacturer, is not guaranteed or endorsed by the publisher.
