# Supplementary material for: Residual Risk of Coronary Atherosclerotic Heart Disease and Severity of Coronary Atherosclerosis Assessed by ApoB and LDL-C in Participants With Statin Treatment: A Retrospective Cohort Study
Source: Front Endocrinol (Lausanne). 2022 Apr 20;13:865863. doi: 10.3389/fendo.2022.865863 (PMC9097510; doi:10.3389/fendo.2022.865863)
Supplement: Supplementary file 1 [file Table_1.docx]

**Supplementary Table 1** Baseline characteristics of participants with statins treatment according to LDL-C concentrations

|  | **LDL-C concentrations（mmol/L）** | | | | |
| --- | --- | --- | --- | --- | --- |
|  | **Total** | **≤1.56** | **1.57-2.30** | **≥2.31** | ***P*_trend_** |
| Baseline characteristics | | | | | |
| Age（years） | 64.98±7.68 | 65.59±7.89 | 64.09±7.18 | 65.26±8.04 | 0.835 |
| Sex |  |  |  |  |  |
| Female | 56（42.7%） | 20（45.5%） | 19（43.2%） | 17（39.5%） | 0.579 |
| Male | 75（57.3%） | 24（54.5%） | 25（56.8%） | 26（60.5%） |  |
| Diabetes |  | | | | |
| No | 83（63.4%） | 25（56.8%） | 26（59.1%） | 32（74.4%） | 0.091 |
| Yes | 48（36.6%） | 19（43.2%） | 18（40.9%） | 11（25.6%） |  |
| Hypertention |  | | | | |
| No | 40（30.5%） | 12（27.3%） | 14（31.8%） | 14（32.6%） | 0.593 |
| Yes | 91（69.5%） | 32（72.7%） | 30（68.2%） | 29（67.4%） |  |
| Smoking status |  | | | | |
| Never smoker | 85（64.9%） | 31（70.5%） | 34（77.3%） | 20（46.5%） | 0.021 |
| Smoker | 46（35.1%） | 13（29.5%） | 10（22.7%） | 23（53.5%） |  |
| Lipids | | | | | |
| TC (mmol/L) | 4.18±1.18 | 3.45±1.43 | 4.01±0.47 | 5.09±0.76 | ＜0.001 |
| TG (mmol/L) | 1.73±2.46 | 2.10±4.15 | 1.48±0.59 | 1.62±0.71 | 0.023 |
| HDL-C (mmol/L) | 1.18±0.31 | 1.12±0.34 | 1.16±0.31 | 1.25±0.26 | 0.006 |
| ApoB (g/L) | 0.81±0.26 | 0.62±0.15 | 0.79±0.21 | 1.02±0.23 | ＜0.001 |
| ApoA1(g/L) | 1.26±0.25 | 1.28±0.24 | 1.27±0.27 | 1.23±0.22 | 0.342 |
| Lp(a)(mg/dL) | 23.94±26.69 | 22.76±28.42 | 21.36±20.51 | 27.77±30.40 | 0.548 |

Data expressed as absolute frequency (%) and mean ± SD

LDL-C was categorized into three groups: low(≤1.56mmol/L)，moderate（1.57-2.30mmol/L），high（≥2.31mmol/L）according to tertiles of LDL-C.

**Supplementary Table 2** Stratified analyses of the associations (RR, 95% CIs) of apoB concentrations with the residual risk of coronary atherosclerotic heart disease in participants with statins treatment

|  | **ApoB concentrations（g/L）** | | | |  |
| --- | --- | --- | --- | --- | --- |
|  | **≤0.66** | **0.67-0.89** | **≥0.90** | ***P*_trend_** | ***P*_interaction_** |
| Age（years） | | | | | 0.484 |
| ＜65 | 1 | 1.35 (1.09, 1.66) | 1.51(1.09,2.08) | 0.034 |  |
| ≥65 | 1 | 1.06 (0.88, 1.29) | 1.25 (0.97, 1.62) | 0.083 |  |
| Sex | | | | | 0.590 |
| Female | 1 | 1.08 (0.88, 1.34) | 1.39 (1.07, 1.79) | 0.014 |  |
| Male | 1 | 1.20 (0.97, 1.47) | 1.23(0.92, 1.65) | 0.259 |  |
| Smoking status | | | | | 0.572 |
| Never smoker | 1 | 1.09(0.94, 1.28) | 1.30(1.03, 1.63) | 0.026 |  |
| Smoker | 1 | 1.29(0.98, 1.68) | 1.36(0.94, 1.96) | 0.184 |  |
| Diabetes | | | | | 0.204 |
| No | 1 | 1.28 (1.10, 1.49) | 1.70(1.28, 2.25) | ＜0.001 |  |
| Yes | 1 | 1.19 (0.92, 1.54) | 1.13 (0.89, 1.44) | 0.435 |  |
| Hypertention | | | | | 0.067 |
| No | 1 | 1.02 (0.81, 1.28) | 1.30 (0.91, 1.88) | 0.175 |  |
| Yes | 1 | 1.25 (1.07, 1.46) | 1.31 (1.05, 1.64) | 0.040 |  |

Adjusted for age, sex, smoking status, diabetes, hypertention, total cholesterol, triglycerides, apolipoprotein A1 and lipoprotein (a), with exception of stratifying factors.

**Supplementary Table 3** Stratified analyses of the associations (RR, 95% CIs) of LDL-C concentrations with the residual risk of coronary atherosclerotic heart disease in participants with statins treatment

|  | **LDL-C concentrations（mmol/L）** | | | |  |
| --- | --- | --- | --- | --- | --- |
|  | **≤1.56** | **1.57-2.30** | **≥2.31** | ***P*_trend_** | ***P*_interaction_** |
| **Age（years）** | | | | | 0.845 |
| ＜65 | 1 | 0.92 (0.74, 1.13) | 0.95(0.65,1.40) | 0.797 |  |
| ≥65 | 1 | 1.04 (0.81, 1.34) | 1.23 (0.81, 1.86) | 0.295 |  |
| **Sex** | | | | | 0.992 |
| Female | 1 | 0.98 (0.79, 1.21) | 1.14 (0.78, 1.68) | 0.493 |  |
| Male | 1 | 0.93 (0.74, 1.17) | 0.96(0.67, 1.36) | 0.858 |  |
| **Smoking status** | | | | | 0.829 |
| Never smoker | 1 | 0.99(0.83, 1.18) | 1.21(0.87, 1.70) | 0.246 |  |
| Smoker | 1 | 1.00(0.72, 1.38) | 0.99(0.68, 1.44) | 0.946 |  |
| **Diabetes** | | | | | 0.230 |
| No | 1 | 0.99 (0.84, 1.17) | 1.14(0.86, 1.51) | 0.385 |  |
| Yes | 1 | 0.93(0.81, 1.08) | 0.83 (0.67, 1.05) | 0.111 |  |
| **Hypertention** | | | | | 0.169 |
| No | 1 | 1.25 (0.98, 1.60) | 1.17 (0.71, 1.93) | 0.293 |  |
| Yes | 1 | 0.95 (0.82, 1.10) | 1.05 (0.83, 1.31) | 0.624 |  |

Adjusted for age, sex, smoking status, diabetes, hypertention, total cholesterol, triglycerides, apolipoprotein A1 and lipoprotein (a), with exception of stratifying factors.

**Supplementary Table 4** OR (95% CIs) for residual risk of coronary atherosclerotic heart disease according to apoB or LDL-C concentrations when using logistic regression

| **ApoB （g/L）** | **≤0.66** | **0.67-0.89** | **≥0.90** | ***P*_trend_** |
| --- | --- | --- | --- | --- |
| Model 1 | 1.00 | 2.01 (0.77, 5.26) | 3.08 (1.19, 7.95) | 0.022 |
| Model 2 | 1.00 | 2.15 (0.78, 5.94) | 3.77 (1.35, 10.50) | 0.012 |
| Model 3 | 1.00 | 3.20 (1.01, 10.19) | 7.34 (1.58, 34.16) | 0.014 |
| **LDL-C（mmol/L）** | **≤1.56** | **1.57-2.30** | **≥2.31** | ***P*_trend_** |
| Model 1 | 1.00 | 0.71(0.28,1.81) | 1.54 (0.64, 3.71) | 0.256 |
| Model 2 | 1.00 | 0.79(0.30, 2.08) | 1.64(0.64, 4.21) | 0.273 |
| Model 3 | 1.00 | 0.90(0.28.2.87) | 1.85(0.33,10.51) | 0.457 |

Model 1：no variables are adjusted.

Model 2：adjusted for age (years), sex (female or male), smoking status (never smoker or smoker), diabetes (no or yes), and hypertention(no or yes).

Model 3：further adjusted for total cholesterol, triglycerides, apolipoprotein A1 and lipoprotein (a).

**Supplementary Table 5** RR (95% CIs) for residual risk of coronary atherosclerotic heart disease according to apoB or LDL-C concentrations when further adjusting the estimated glomerular filtration rate

| **ApoB （g/L）** | **≤0.66** | **0.67-0.89** | **≥0.90** | ***P*_trend_** |
| --- | --- | --- | --- | --- |
| Model 1 | 1.00 | 1.11 (0.96, 1.29) | 1.20 (1.04, 1.38) | 0.017 |
| Model 2 | 1.00 | 1.11 (0.97, 1.27) | 1.21 (1.06, 1.39) | 0.008 |
| Model 3 | 1.00 | 1.17 (1.02, 1.34) | 1.32 (1.09, 1.61) | 0.008 |
| **LDL-C（mmol/L）** | **≤1.56** | **1.57-2.30** | **≥2.31** | ***P*_trend_** |
| Model 1 | 1.00 | 0.95 (0.82,1.10) | 1.08 (0.93, 1.25) | 0.265 |
| Model 2 | 1.00 | 0.96(0.84, 1.11) | 1.08(0.93, 1.26) | 0.294 |
| Model 3 | 1.00 | 0.98(0.84.1.15) | 1.08(0.84,1.40) | 0.533 |

Model 1：no variables are adjusted.

Model 2：adjusted for age (years), sex (female or male), smoking status (never smoker or smoker), diabetes (no or yes), and hypertention(no or yes).

Model 3：further adjusted for total cholesterol, triglycerides, apolipoprotein A1, lipoprotein (a) and eGFR.

**Supplementary Table 6** RR (95% CIs) for residual risk of coronary atherosclerotic heart disease according to apoB or LDL-C concentrations after selecting individuals with higher syntax scores

| **ApoB （g/L）** | **≤0.66** | | **0.67-0.89** | **≥0.90** | ***P*_trend_** |
| --- | --- | --- | --- | --- | --- |
| Model 1 | 1.00 | | 1.14 (0.94, 1.37) | 1.18 (0.98, 1.42) | 0.086 |
| Model 2 | 1.00 | | 1.14 (0.95, 1.37) | 1.23 (1.02, 1.48) | 0.031 |
| Model 3 | 1.00 | | 1.23 (1.02, 1.48) | 1.43 (1.10, 1.86) | 0.011 |
| **LDL-C（mmol/L）** | | **≤1.56** | **1.57-2.30** | **≥2.31** | ***P*_trend_** |
| Model 1 | 1.00 | | 0.95 (0.79,1.14) | 1.01 (0.85, 1.20) | 0.836 |
| Model 2 | 1.00 | | 0.96(0.80, 1.15) | 1.03(0.85, 1.24) | 0.723 |
| Model 3 | 1.00 | | 1.02(0.80.1.30) | 1.09(0.75,1.59) | 0.645 |

Model 1：no variables are adjusted.

Model 2：adjusted for age (years), sex (female or male), smoking status (never smoker or smoker), diabetes (no or yes), and hypertention(no or yes).

Model 3：further adjusted for total cholesterol, triglycerides, apolipoprotein A1, lipoprotein (a) and eGFR.
